# Enhanced YOLOv8-seg-SPDConv for accurate Schatzker classification of tibial plateau fractures

**DOI:** 10.3389/fmed.2026.1843762

**Published:** 2026-09-22

**Authors:** Haoteng Wang, Zhiheng Gao, Heting Xiao, Xuwei Ling, Haowen Lu, Yu Chen, Minbo Jian, Zheming Shen, Quan Zhou, Peng Yang, Tao Liu, Yusen Qiao

**Affiliations:** Department of Orthopaedics, The First Affiliated Hospital of Soochow University, Suzhou, China

**Keywords:** computer-aided diagnosis (CAD), mean average precision (MAP), medical image analysis, Schatzker classification, SPDConv, tibial plateau fractures, YOLOv8-seg

## Abstract

**Background:**

Tibial plateau fractures are typically caused by direct or indirect violent forces, often resulting in collapse fractures of the medial or lateral tibial plateau. Accurate diagnosis and classification of tibial plateau fractures are of significant importance for determining appropriate treatment plans. However, manual Schatzker classification is subject to considerable inter-observer variability, particularly among junior physicians and non-specialist clinicians, often resulting in suboptimal accuracy and prolonged decision-making time in high-throughput emergency settings.

**Objective:**

This study aimed to develop and validate a lightweight YOLOv8n-seg-SPDConv model for the joint instance segmentation and Schatzker classification of tibial plateau fractures using thin-slice axial CT images from 552 unique patients. Specifically, by integrating the Space-to-Depth Convolution (SPDConv) module, we sought to preserve sub-2 mm cortical disruptions while reducing model complexity and suggesting potential for future deployment on resource-constrained platforms. Using a hold-out test set benchmarked against a senior orthopedic surgeon's consensus standard, we systematically evaluated the model's diagnostic performance, inference efficiency (226 FPS), and interpretability via Gradient-weighted Class Activation Mapping (Grad-CAM), with the ultimate goal of serving as a reliable real-time clinical adjunct that supports, rather than supplants, clinicians in emergency and perioperative decision-making.

**Methods:**

From October 2017 to January 2026, a dataset comprising 552 axial CT slices from unique patients was collected. Each image was independently annotated into six clinically relevant categories according to the Schatzker classification. To prevent data leakage, dataset partitioning was strictly performed prior to augmentation using an 8:2 patient-wise split. The YOLOv8n-seg network was employed for joint segmentation and classification, with multiple preprocessing techniques applied to ensure data consistency and enhance model performance.

**Results:**

The baseline YOLOv8n-seg achieved mAP50 values of 0.941, 0.956, 0.861, 0.818, 0.916, and 0.869 for Schatzker types I–VI, respectively. The improved YOLOv8n-seg-SPDConv yielded mAP50 of 0.928, 0.952, 0.929, 0.859, 0.925, and 0.893 for the six subtypes, with an overall mAP50 increasing from 0.893 to 0.914. Notably, the proposed model reduced parameters from 3.01 M to 2.64 M and FLOPs from 8.1 G to 7.3 G, while increasing inference speed from 188 to 226 FPS, meeting real-time requirements for edge deployment. Grad-CAM visualization confirmed that the model focused its attention on clinically meaningful regions, including fracture fissures and articular depressions, rather than background artifacts.

**Discussion:**

This deep learning-based classification method provides efficient and reliable automated assessments to assist and augment manual evaluation by clinicians, with the potential to help reduce interobserver variability among junior physicians during emergency and perioperative decision-making. It shows promise as a tool that may help mitigate interobserver variability and support junior physicians in emergency and primary care settings. While marginal performance variations were observed in simple linear fractures, the model achieved substantial gains in complex, low-contrast subtypes. These findings highlight its value as a complementary clinical decision support tool, with the potential to support and streamline diagnostic workflows rather than replace expert judgment, ultimately contributing to improved patient outcomes.

## 1 Introduction

Tibial plateau fractures are a clinically prevalent type of fracture, representing one of the most complex and common intra-articular fractures encountered in orthopedic trauma surgery. They account for approximately 1% of all adult fractures, with an annual incidence of 10.3 per 100,000 patients (1), and the incidence has shown an upward trend in recent years (2, 3). Clinical manifestations typically include knee joint swelling, pain, and limited mobility. Improper management often leads to complications such as malunion, post-traumatic arthritis, and knee stiffness (4, 5), causing significant distress to patients. Research into the treatment methods and postoperative management of tibial plateau fractures is crucial for improving patients' quality of life.

To effectively treat tibial plateau fractures, it is first essential to understand the characteristics of different fracture types, which necessitates the accurate classification of the various tibial plateau fractures (6). Currently, there are at least 38 different classification systems (7). Among these, widely recognized and utilized systems include the Schatzker classification, AO/OTA classification, and the three-column classification (8, 9). The Schatzker classification is concise, intuitive, easy to grasp, and demonstrates high reliability and reproducibility (10). Based on the condition of the tibial articular surface, Schatzker classified tibial plateau fractures into six types. Type I is a pure lateral split fracture, commonly seen in young individuals with good bone density; Type II involves a lateral split combined with articular surface depression and is the most common among the six types; Type III is characterized by pure central depression of the lateral plateau without a lateral split; Type IV involves the medial plateau, often combined with knee dislocation and vascular injury, and has a poor prognosis; Type V is a bicondylar fracture involving injury to both condyles; Type VI is a bicondylar fracture with separation of the metaphysis and diaphysis, characterized by high energy, comminution, and severe soft tissue injury (6).

At the clinical level, although the Schatzker classification system is widely adopted in clinical practice, its manual assessment is inherently complex, particularly for junior physicians. First, substantial overlap exists among the fracture patterns of different subtypes—particularly between Schatzker types II and III, both of which involve tibial plateau depression. Visually distinguishing whether peripheral cortical splitting is present poses a significant challenge. Moreover, type IV fractures often extend toward the intercondylar eminence, where the fracture lines can be readily confounded with normal osseous structures on axial CT images. Second, key diagnostic features are frequently subtle: cortical disruptions in type I fractures may measure less than 2 mm, while the central depression characteristic of type III lesions is often difficult to differentiate from “pseudo-depression” artifacts induced by the partial volume effect, placing stringent demands on image interpretability. Third, interobserver reliability remains a major concern. Studies indicate that even among experienced orthopedic trauma surgeons, the kappa values for Schatzker classification typically remain at a moderate level. In cohorts involving junior surgeons or multicenter studies, discrepancies become even more pronounced due to inconsistent criteria for defining “depression depth” and “displacement magnitude.” Finally, classification outcomes are heavily dependent on clinician experience: novice surgeons often struggle to identify occult fracture lines and are prone to missed or erroneous diagnoses under the fatigued conditions typical of emergency settings. This diagnostic inconsistency, which stems from the combined effects of anatomical complexity, imaging limitations, and human factors, directly compromises surgical planning and postoperative outcome evaluation. To address these clinical challenges, deep learning-based approaches have been explored in recent years.

At the methodological level, several deep learning studies have attempted to automate Schatzker classification, yet critical gaps remain. Sato et al. (15) demonstrated that deep learning models can significantly improve the diagnostic accuracy of resident physicians in detecting hip fractures, while Lindsey et al. (17) developed a deep neural network that outperformed radiologists in fracture detection on chest radiographs. With specific regard to tibial plateau fractures, several studies have attempted to automate Schatzker classification using deep learning. For instance, Van der Gaast et al. (11) employed deep learning frameworks such as ResNet to analyze knee radiographs, enabling fracture detection and Schatzker classification. However, their model was limited to image-level classification and lacked the capability for precise localization of fracture foci. On the other hand, Cai et al. (12) utilized a 3D U-Net architecture to perform semantic segmentation of tibial plateau fracture regions in knee CT scans, successfully constructing three-dimensional morphological atlases of fractures. Nevertheless, this model focused primarily on morphometric quantification and could not directly output clinically required Schatzker subtype labels; manual interpretation remained necessary to finalize the classification.

The application of CT technology has significantly improved the accuracy and reliability of tibial plateau classification systems (7, 13, 14). However, traditional manual classification is susceptible to subjectivity and has certain limitations in terms of classification accuracy and reliability. Factors such as fatigue and mental stress can easily lead clinicians to misinterpret images, resulting in classification errors and subsequent treatment mistakes (15). Such errors are likely to have serious consequences, including skeletal malunion and osteonecrosis (16). To reduce the occurrence of such situations, an imaging diagnostic model based on extensive clinical data and capable of accurate classification is needed. Applying deep learning technology to clinical image diagnosis can quickly and efficiently identify abnormal areas in clinical images, providing significant convenience for clinicians in their diagnostic work (17–19) In recent years, the application of deep learning technology in clinical practice has become increasingly widespread, and it has been utilized in the diagnosis and treatment of orthopedic conditions such as lumbar disc herniation, osteoporosis, and distal radius fractures (20–23) Some studies have shown that networks like U-Net and CNN possess good denoising performance and excel in segmentation tasks (18, 20, 24, 25). In comparison, the YOLOv8-seg network offers higher computational speed and accuracy, lower computational complexity, and the advantages of being lightweight and efficient (22, 26).

In the domain of object detection, the YOLO family has emerged as a mainstream framework in medical image analysis due to its superior real-time performance. Liu et al. (27) employed YOLOv5 to detect long-bone fractures, maintaining high frame rates alongside robust detection sensitivity. Similarly, Chen et al. (28) adapted YOLOv7 for pelvic fracture identification on CT scans, validating the feasibility of single-stage detectors in complex fracture scenarios. Nevertheless, most existing YOLO-based studies focus primarily on “fracture presence detection” or coarse localization, with few investigations systematically optimized for the nuanced six-type Schatzker classification task.

Beyond the limitations of existing AI frameworks, a fundamental technical bottleneck persists in the feature extraction pipeline itself. Notably, while the vast majority of relevant studies utilize routine axial CT as input, few have explicitly addressed two core challenges: preservation of small-target features and mitigation of class imbalance. Conventional strided convolutions in standard YOLO architectures inevitably discard fine-grained features—such as subtle fracture lines—during downsampling, which is particularly disadvantageous for Schatzker classification that relies heavily on the discrimination of minute cortical disruptions and articular depressions. SPDConv, as an information-loss-free downsampling strategy, has demonstrated promise in remote sensing and small-object detection; however, its application to orthopedic medical imaging—and specifically to TPF classification—remains unreported. Meanwhile, although previous studies have highlighted the diagnostic value of deep learning in fracture detection, limited attention has been paid to its potential role in reducing inter-observer variability among junior clinicians and standardizing multicenter research protocols.

In summary, despite encouraging progress in deep learning–based fracture diagnosis, critical gaps remain in the automation of Schatzker classification for TPFs: (1) a lack of integrated frameworks capable of simultaneous detection, segmentation, and six-type classification; (2) inadequate retention of subtle fracture features due to conventional downsampling strategies, compromising classification accuracy; (3) insufficient systematic optimization for class imbalance and small-target recognition; and (4) a disconnect between algorithmic development and practical clinical needs, such as trainee education and multicenter standardization. To address these limitations, this study introduces SPDConv into the YOLOv8-seg architecture for the first time, proposing a YOLOv8n-seg-SPDConv model. By reducing model complexity while preserving critical fracture texture features, our approach aims to achieve high-precision, lightweight Schatzker classification and provide a reliable tool for standardized clinical teaching and multicenter diagnostic workflows.

Accordingly, to address the critical gap in standardized Schatzker classification—particularly the notable inter-observer variability among junior clinicians and the lack of consistent subtyping in multicenter research—this study aims to develop and validate an improved YOLOv8n-seg-SPDConv model. While experienced surgeons can readily distinguish fracture patterns using multi-modal imaging, the nuanced differentiation between subtypes remains challenging for trainees and in high-throughput emergency settings. By integrating the SPDConv module, we preserve subtle radiographic features to enhance the model's educational utility as a digital teaching aid for resident training. Furthermore, we evaluate the model's capacity to serve as a standardized reference tool to harmonize subtyping across multicenter clinical studies, thereby facilitating reliable comparative effectiveness research and supporting consistent decision-making in primary care and emergency departments where immediate expert consultation may be unavailable.

## 2 Methods

### 2.1 Study subjects

The dataset comprises 552 CT images of tibial plateau fractures collected from our hospital and affiliated institutions between October 2017 and January 2026, all obtained from patients diagnosed with tibial plateau fractures. Each CT image is from a distinct patient.

This single-center retrospective study was approved by the Ethics Committee of the First Affiliated Hospital of Soochow University [Approval No. (2026)LSNP941]. In view of the retrospective design and full anonymization of imaging data prior to analysis, the requirement for individual informed consent was waived by the Ethics Committee.

### 2.2 Principle of YOLOv8 object detection algorithm

The YOLOv8-seg network comprises a backbone network, a neck network, and a head network. Building on the original C3 module of YOLOv5, it integrates ideas from ELAN to construct a new C2f module. This enhancement increases the richness of gradient information the model obtains during the feature extraction stage. The SPPF module replaces the Spatial Pyramid Pooling, or SPP, module, thereby avoiding distortion issues caused by cropping and scaling image regions. It maintains high-speed computation while optimizing model performance. The YOLOv8-seg network offers versions of progressively larger model sizes: n, s, m, l, and x. Considering the potential for future deployment on resource-constrained platforms, this study selects the lightweight YOLOv8n-seg model.

The backbone network of YOLOv8-seg consists of Conv modules, C2f modules, and an SPPF module. Drawing on the Darknet structure, it employs four serial stacks of Conv and C2f modules for feature extraction from input images. The extracted feature maps show a trend of gradually decreasing size and increasing channel count. The Conv module integrates convolutional layers, batch normalization known as BN, and the SiLU activation function. It is primarily used for feature extraction and adjusting the number of channels in feature maps. The C2f module performs deep feature extraction by splitting the feature map, serially connecting bottleneck layers, and then concatenating feature maps. The SPPF module performs feature extraction and encoding at different scales. It does this by concatenating the results of three max-pooling operations on the input feature map, followed by convolution. By combining advanced existing network structures, the backbone network achieves powerful feature extraction capabilities.

The neck network is composed of C2f modules, Upsample operations, and Concat modules. It fuses features from different scales. Unlike the backbone, the C2f modules in the neck have different input and output channel counts. Their output channel number matches the size of the feature map at the corresponding layer in the backbone. Additionally, each C2f here contains only one bottleneck layer, which itself has no internal residual connections. Through its upsampling, downsampling, and feature extraction structure, the network enhances its ability to perceive features at various scales.

The YOLOv8 head network adopts the decoupled head design from YOLOX. It separately computes the localization loss and classification loss for three input feature maps of different sizes. This process yields predicted locations and classification results for targets at various scales. The YOLOv8-seg detection head adds a mask segmentation branch, called the segmentation head, to the conventional object detection head. Its structure is inspired by the YOLACT network. It extracts prototype masks, referred to as Proto, from the largest feature map. It also extracts mask coefficients, or coeff, from the prediction boxes at each scale. Finally, it generates the semantic masks by linearly combining the prototype masks and mask coefficients. Therefore, the YOLOv8-seg network combines the three tasks of object classification, bounding box localization, and instance segmentation. This gives the model efficient segmentation capabilities while maintaining good real-time performance and segmentation accuracy. These innovations in YOLOv8 demonstrate its considerable potential and broad application prospects in medical image detection (29). Particularly for Schatzker classification in medical imaging, the high precision and efficiency of YOLOv8 provide strong support for improving diagnostic accuracy. The YOLOv8-seg model facilitates simultaneous detection and segmentation, enabling not only the classification of fracture types but also the precise delineation of fracture sites. Consequently, the model effectively localizes injury regions by generating accurate segmentation masks around fracture fissures.

Although the YOLO series has evolved to v11, YOLOv8n-seg was selected as the baseline. Firstly, YOLOv8 offers a mature instance segmentation architecture, while newer variants show instability in small-sample CT tasks. Secondly, YOLOv8n optimally balances overfitting control and feature extraction. To empirically validate this choice, we conducted a comparative experiment among YOLOv5n-seg, YOLOv8n-seg, and YOLO11n-seg (Table 1). Results demonstrated that YOLOv8n-seg achieved superior performance across all metrics: Mask Precision (P) of 0.848, Recall (R) of 0.842, mAP0.5 of 0.893, and mAP0.5:0.95 of 0.82, outperforming YOLOv5n-seg (*P* = 0.821, R = 0.825, mAP0.5 = 0.877) and YOLO11n-seg (*P* = 0.827, R = 0.835, mAP0.5 = 0.889). This empirically confirms that YOLOv8n-seg provides the best trade-off between accuracy and stability for our study.

**Table 1:** Performance Comparison of Different YOLO Segmentation Models .

model	Mask(P)	R	mAP0.5	mAP0.5:0.95
YOLOv5n-seg	0.821	0.825	0.877	0.813
YOLOv8n-seg	0.848	0.842	0.893	0.82
YOLO11n-seg	0.827	0.835	0.889	0.807

### 2.3 Data preprocessing

During preprocessing for input into YOLOv8, the images underwent a series of adjustments. This included standardizing them to a uniform size and resolution, followed by conversion to grayscale. The grayscale conversion reduces the number of image channels, thereby improving image processing efficiency.

### 2.4 Dataset construction

Under the direct mentorship of a senior orthopedic surgeon (Associate Chief Physician) with over 10 years of specialized experience in tibial plateau fracture management, a medical intern annotated the CT images according to the six Schatzker types using the Labelme annotation software (Figure 1). Unlike conventional parallel independent labeling, this mentorship model ensured the accurate real-time interpretation of subtle imaging features. To establish a robust clinical ground truth, a rigorous consensus protocol was adopted. All annotations were initially guided by the senior surgeon. Subsequently, to eliminate individual bias, a random subset of 20% of the dataset was selected for a blinded secondary review by a second senior specialist. Any discrepancies identified during this review were resolved through joint discussion until a unanimous consensus diagnosis was reached. Consequently, the finalized dataset reflects a unified expert consensus, serving as the gold standard for evaluating model performance.

**Figure 1:**
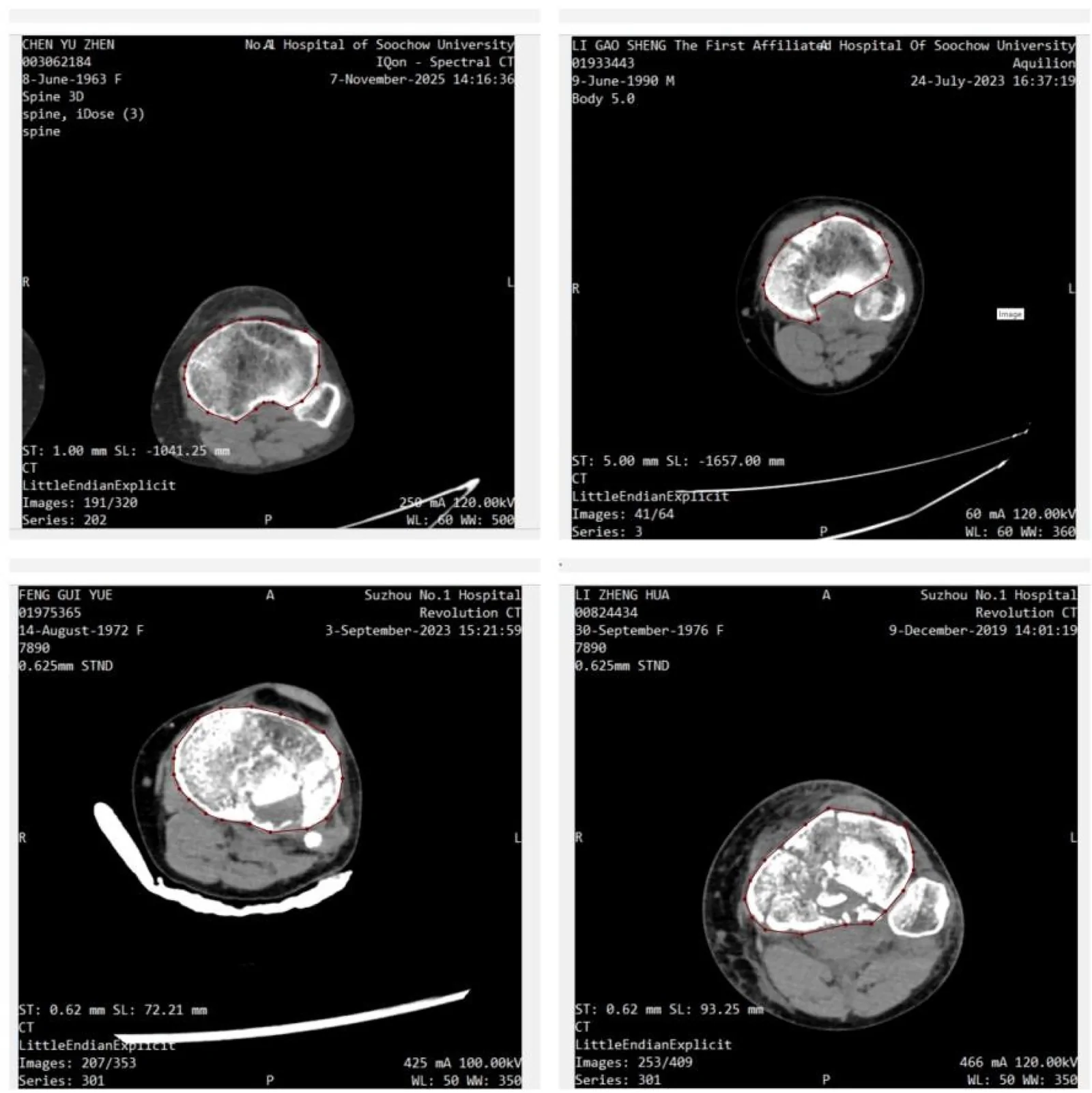
Labelme annotation.

Among the 552 CT images from unique patients, the distribution comprised 114 Type I, 123 Type II, 94 Type III, 71 Type IV, 88 Type V, and 62 Type VI fractures. To prevent inflated model metrics caused by cross-contamination between augmented and original data, dataset partitioning was strictly performed prior to augmentation using an 8:2 patient-wise split. Specifically, 442 patients (80%) were allocated to the training set and 110 patients (20%) to the hold-out test set. Gaussian noise was then applied exclusively to the training set, generating one augmented counterpart for each original training image, while the test set retained only unaugmented original images. Consequently, the training set comprised 884 images (442 original + 442 augmented), and the test set consisted of 110 original images. No augmented image appeared in the test set, and no original image and its augmented counterpart were distributed across different subsets. This augmentation strategy enhances the model's detection capability under low signal-to-noise ratio conditions, as illustrated in Figure 2, thereby improving its overall robustness.

**Figure 2:**
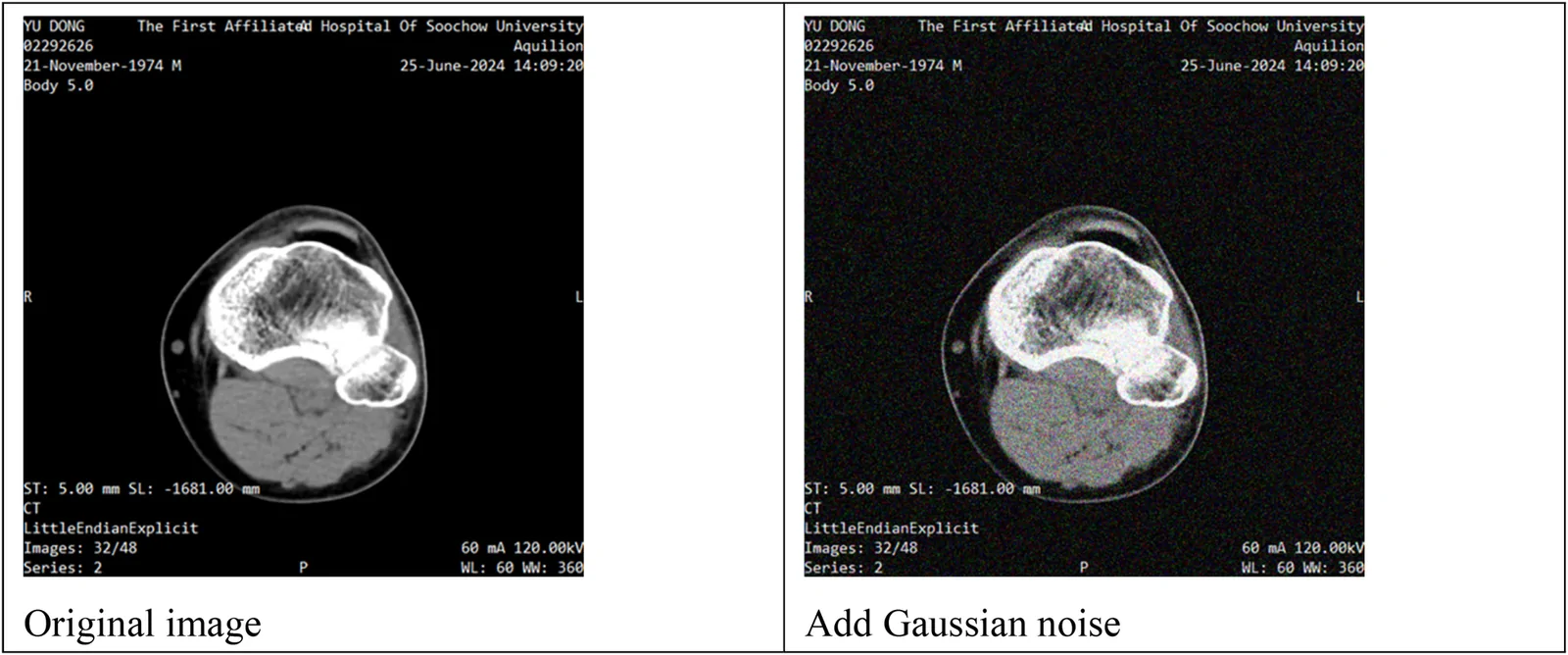
Image enhancement comparison.

During data augmentation, we employed random horizontal flipping. Crucially, this operation was applied to individual axial CT slices rather than to the entire three-dimensional volume. Given the anatomical constraints of tibial plateau fractures, it is important to note that the spatial relationship between the fracture fragment and the tibial spine remains anatomically consistent post-flipping in the axial plane. For instance, in a Schatzker type IV fracture, the medial location relative to the midline is preserved; the transformation merely simulates the mirror-image presentation of left vs. right limbs. This strategy enables the model to learn orientation-invariant features, thereby enhancing generalizability across lateralities without introducing anatomically erroneous samples.

Due to constraints related to the collection of retrospective clinical data and the need to ensure annotation consistency, all deep learning model inputs and manual annotations in this study were based exclusively on thin-slice axial CT images, without incorporating multiplanar reconstructions such as sagittal or coronal views. The core imaging features of each Schatzker subtype on axial CT are defined as follows: Type I is characterized by focal cortical discontinuity of the lateral tibial plateau, with a longitudinal radiolucent fracture line and no articular surface depression; the typical sign is best observed on the axial slice where the fracture line is most clearly demonstrated, requiring careful identification of subtle cortical interruptions smaller than 2 mm. Type II presents with both cortical disruption of the lateral plateau and articular depression, where collapsed bone fragments adjacent to the fracture line appear as areas of increased density on axial images; the extent of depression should be assessed by comparing with the contralateral intact articular surface. Type III manifests as a central depression of the lateral plateau without peripheral cortical splitting; on axial images, it appears as a localized step-off of the subchondral cortex, which must be differentiated from pseudodepression caused by partial volume averaging. Type IV involves cortical rupture of the medial plateau, with fracture lines often extending toward the intercondylar eminence, and axial images reveal morphological distortion of the medial plateau along with displaced fragments. Type V shows independent fracture lines involving both the medial and lateral plateaus, while continuity between the metaphysis and diaphysis is preserved on axial views. Type VI is characterized by bicondylar fractures combined with metaphyseal–diaphyseal dissociation, which is clearly depicted on axial images as displacement at the metaphysis–diaphysis junction, frequently accompanied by bone impaction and surrounding soft tissue swelling.

The decision to use only axial images was based on three main considerations. First, in routine clinical practice—particularly in primary hospitals and emergency settings—axial sequences are the default storage format for CT data; omitting multiplanar reconstruction reduces preprocessing complexity and aligns more closely with real-world deployment scenarios. Second, axial images offer consistent spatial resolution and fixed slice thickness, minimizing feature interference caused by variations in reconstruction algorithms and interslice spacing, thereby facilitating stable learning of fracture texture patterns by the model. Third, existing studies have demonstrated that experienced orthopedic surgeons can accurately determine the majority of Schatzker classifications using axial images alone; thus, this approach does not substantially compromise clinical feasibility, while supporting lightweight model deployment in resource-limited environments.

Notably, while the dual-expert consensus protocol minimizes individual bias, we did not quantitatively assess inter-observer agreement prior to consensus, as independent pre-consensus classifications were not retained separately.

### 2.5 A YOLOv8-seg-based improved algorithm for schatzker type detection

To enhance the model's localization accuracy in Schatzker-type CT images, improve the precision of specific category judgment, reduce computational load and the number of parameters, and boost overall operational efficiency, key improvements were made based on the YOLOv8-seg model framework. This led to the proposed YOLOv8-seg-SPDConv model, whose structure is depicted in Figure 3. The improvement involves replacing the traditional convolutional downsampling (Conv) modules in both the backbone and neck networks of YOLOv8 with SPDConv modules.

**Figure 3:**
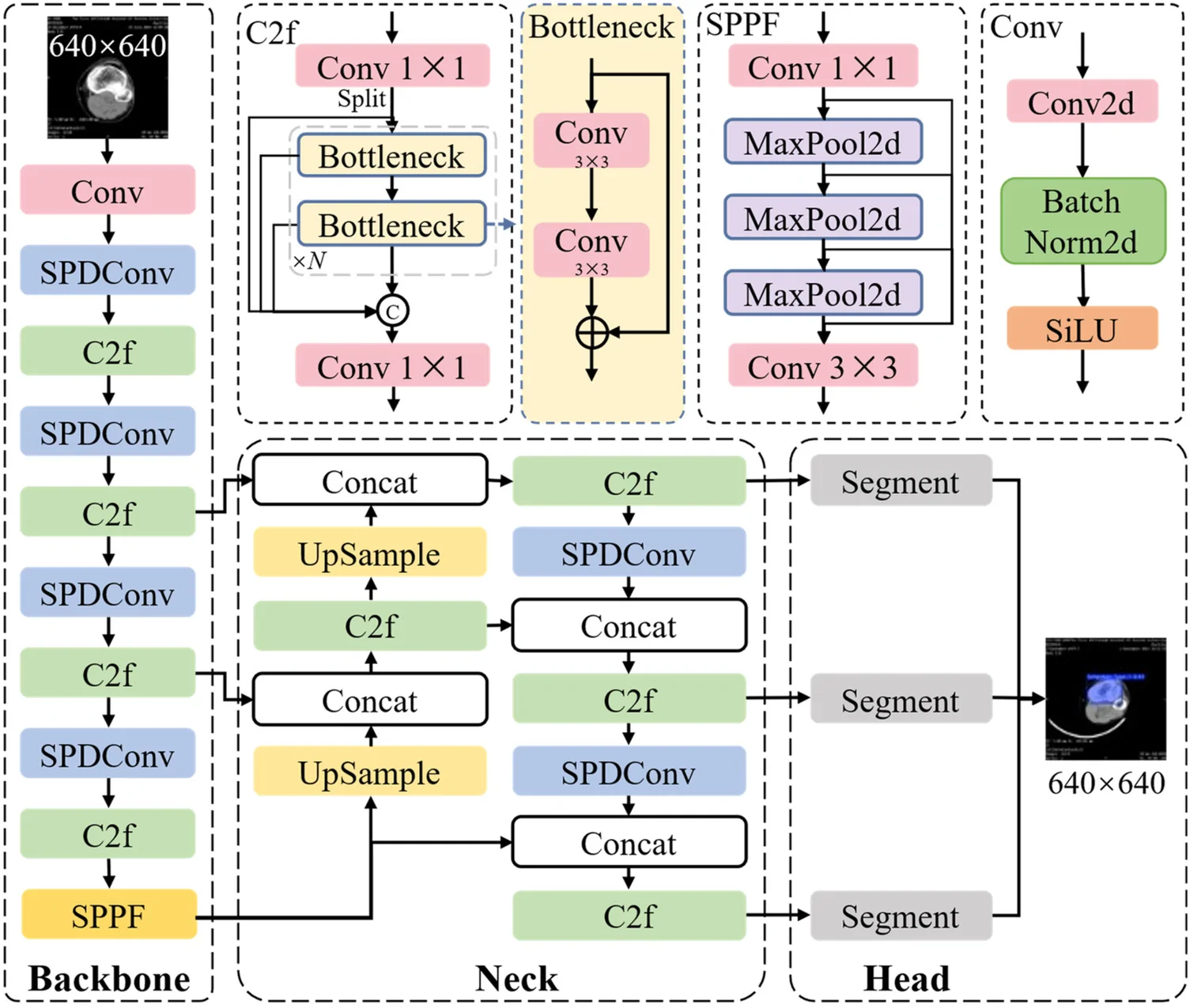
Architecture of the proposed YOLOv8n-seg-SPDConv model. Conventional stride-2 convolutional downsampling modules in the Backbone (two locations) and Neck (one location) were substituted with SPDConv components, as detailed in Section 2.5. The internal structure of the SPDConv module is illustrated in Figure 4.

Specifically, three stride−2 convolutional downsampling layers were replaced with SPDConv modules: two in the backbone network (after the second and third C2f stages, corresponding to the transitions from 80 × 80 to 40 × 40, and from 40 × 40 to 20 × 20, respectively) and one in the neck network (during the top-down pathway, where feature maps are downsampled from 20 × 20 to 10 × 10). These three positions were selected because they govern the transitions between consecutive feature pyramid levels, where the risk of discarding fine-grained fracture features—such as subtle cortical disruptions and articular depressions—is highest. The remaining convolutional layers, including those with stride=1 for feature extraction and channel adjustment, were kept unchanged to maintain model efficiency.

The SPDConv module comprises a Space-to-Depth transformation (scale=2) followed by a 1 × 1 non-strided convolution for channel reduction. This substitution preserves fine-grained spatial information that conventional strided convolutions would irreversibly discard, while maintaining computational efficiency. The internal workflow of the SPDConv module is detailed in Figure 4.

**Figure 4:**
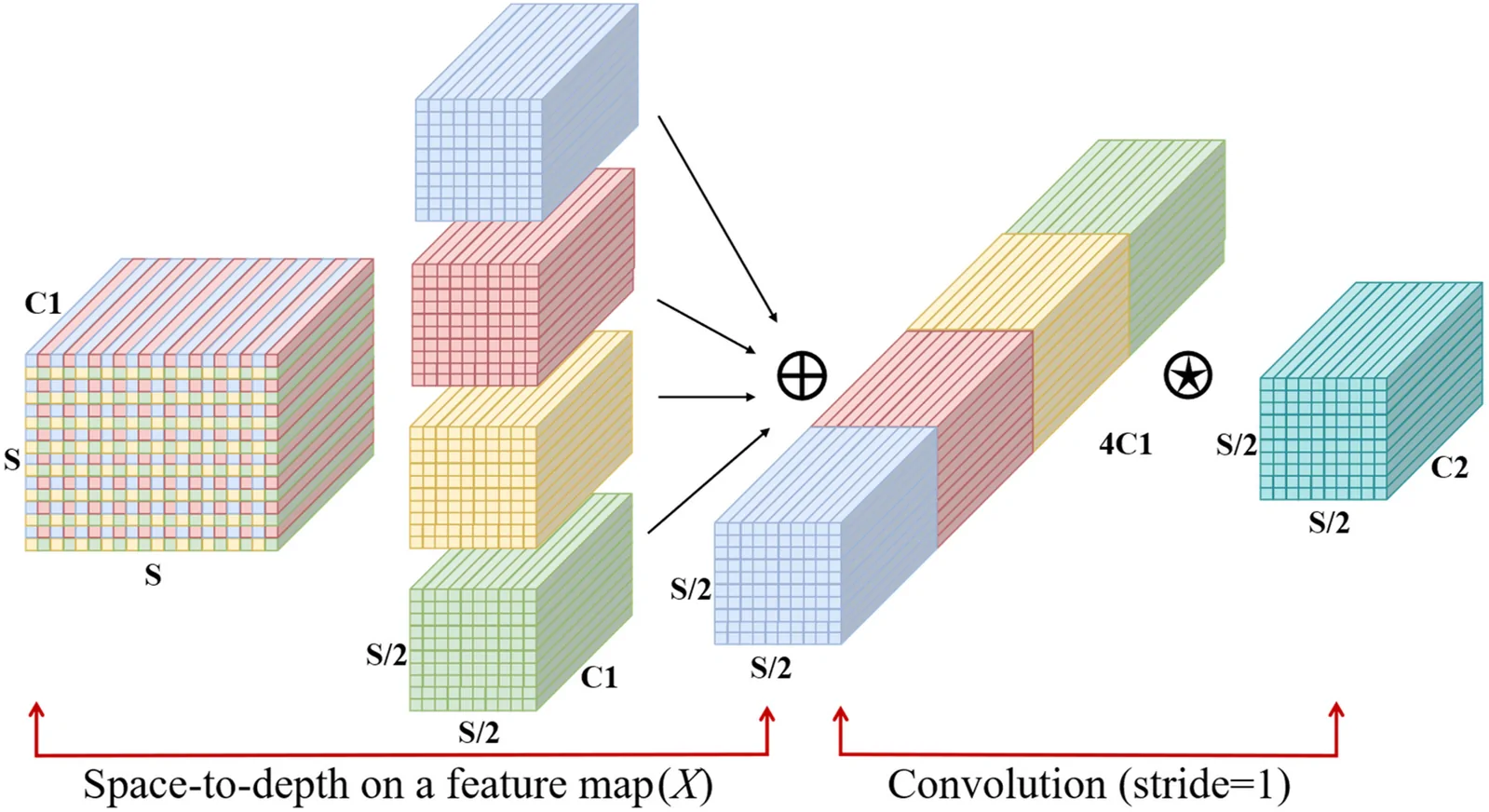
Schematic diagram of the SPDConv module.

In the Schatzker-type CT image samples used in this study, there exist small targets that are crucial for differentiation. The traditional downsampling operation in the Conv module employs a 3 × 3 convolution with a stride of 2 for feature extraction. This approach not only increases the model's parameter count and computational load but also often fails to capture the features of small targets, leading to feature loss and missed detections. To address the problem of losing fine-grained target information, this study introduces the Space-to-Depth Convolution (SPDConv) module into the YOLOv8n model, replacing the Conv downsampling modules in both the backbone and neck networks. This modification effectively mitigates the loss of small target information during the feature extraction process. The workflow of the SPDConv module is illustrated in Figure 4.

As shown in Figure 4, the SPDConv module consists of a Space-to-Depth (SPD) layer and a non-strided convolutional layer with a stride of 1. Assuming the input feature map has dimensions S × S × C₁, it first undergoes the SPD layer. This layer partitions the input feature map into scale × scale number of sub-feature maps through a process akin to interval sampling, effectively downsampling it. To prevent the loss of fine-grained target feature information caused by strided convolutions, the scale value in this study is set to 2. Next, the resulting sub-feature maps are concatenated along the channel dimension. This produces a new feature map whose spatial dimensions are reduced by a factor of 2, and whose channel count is increased by a factor of 4. This operation transforms spatial information into channel information, thereby preserving the complete information from the image. Finally, dimensionality reduction is performed via a 1 × 1 non-strided convolution, yielding a feature map with dimensions of S/2 × S/2 × C₂.The Space-to-Depth transformation avoids the irreversible information loss inherent in stride-2 convolutions, while the subsequent 1 × 1 convolution mitigates the slight latency potentially introduced by channel concatenation.

### 2.6 Experimental environment

This study proposes a novel detection method called YOLOv8-seg-SPDConv, based on the YOLOv8-seg deep learning network. The experiments were conducted on a system running Ubuntu 20.04, equipped with an NVIDIA 3090 24GB graphics card. The virtual environment was configured with Python version 3.10, PyTorch version 2.1.0, and CUDA version 11.8.

The implementation used the official Ultralytics YOLOv8n-seg model, version 8.2.103. Image augmentation techniques, such as Mosaic augmentation, horizontal flip, scaling, and translation, were employed to enhance the model's robustness. The configured hyperparameters are listed in the Table 2 below.

**Table 2:** Experimental Parameter Settings.

Hyperparameter	Value	Hyperparameter	Value
epoch	300	optimizer	SGD
imgsz	640 × 640	lrf	0.01
batch size	16	lr0	0.01
workers	8	momentum	0.937

### 2.7 Evaluation Metrics

The experiments in this study employ Precision (P), Recall (R), and mean average precision (mAP) to evaluate the detection performance of the YOLOv8-seg-SPDConv model.

Precision (P) represents the proportion of actual positive samples among all samples predicted as positive. Its calculation formula is as follows:

$$P=\frac{TP}{TP+FP}$$

In the formula: TP (True Positive) represents the number of correctly classified positive samples, i.e., targets marked as fractures that are detected as fractures; FP (False Positive) represents the number of incorrectly detected negative samples, i.e., targets not marked as fractures that are detected as fractures.

Recall (R) is calculated as follows:

$$R=\frac{TP}{TP+FN}$$

In the formula: FN (False Negative) represents the number of missed positive samples, i.e., targets marked as fractures that were not detected as fractures.

Mean average precision (mAP) employs mAP50 (with an IoU threshold of 0.5) and mAP50:95 (with IoU thresholds ranging from 0.5 to 0.95) as evaluation metrics. The calculation formula for mAP is as follows:

$$mAP=\frac{1}{N}\sum_{i=1}^{N}AP_{i}$$

In the formula: N is the number of detection target categories; for this study, N is set to 6. AP_i represents the average precision for category i, and its calculation formula is:

$$AP_{i}=\int_{0}^{1}P_{i}\cdot R_{i}$$

In the formula: P_i and R_i represent the precision and recall for the detected category i, respectively.

To address the class imbalance across the six Schatzker subtypes, a class-weighted loss function was employed during training. The weight wc for each class c was calculated as the inverse of its sample frequency, normalized by the number of classes:

$$\;\;\;\;\;\;\;\;w_{c}=\frac{N}{n_{c}\;\times C}$$

where *N* = 442 is the total number of training samples, $n_{c}$ is the sample count for class c in the training set, and C = 6 is the number of classes. Based on the training set distribution (Type I: 91, Type II: 98, Type III: 75, Type IV: 57, Type V: 70, Type VI: 51), the resulting class weights were: Type I: 0.646, Type II: 0.599, Type III: 0.784, Type IV: 1.037, Type V: 0.837, Type VI: 1.188. These weights were applied to the classification loss term during training, ensuring that minority classes (particularly Types IV and VI) contributed more significantly to the loss gradient, thereby reducing the model's bias toward majority classes.

## 3 Results

Table 3 summarizes the baseline characteristics of the dataset used in this study. The Precision, recall, mAP50, and mAP50-95 of the YOLOv8n-seg model and the improved YOLOv8n-seg-SPDConv model are shown in Table 4. The original YOLOv8n-seg model achieved an overall mean average precision of 89.3% for Schatzker classification. The improved YOLOv8n-seg-SPDConv model increased the overall mAP50 to 91.4%, demonstrating enhanced performance in Schatzker classification. Specifically, compared to the original model, the improved model's mAP50 for the six different types—Type I, II, III, IV, V, and VI—changed by −1.3%, −0.4%, +6.8%, +4.1%, +0.9%, and +2.4%, respectively. Although the precision for Type I and Type II slightly decreased, the precision for the remaining four types showed varying degrees of improvement, the improved model achieved an overall mAP50 increase from 89.3% to 91.4%, demonstrating that the integration of SPDConv generally enhances the accuracy of Schatzker classification. Overall, the improved model effectively enhanced the accuracy of Schatzker classification.

**Table 3:** Baseline characteristics of the dataset.

Characteristic	Value
Total images	552
Unique patients	552
Collection period	October 2017 – January 2026
Imaging modality	Axial CT (thin-slice, slice thickness ≤ 1.5 mm)
Annotation tool	Labelme
Annotation supervision	Senior orthopedic surgeon (Associate Chief Physician, >10 years experience)
Consensus verification	20% random subset, blinded secondary review by second senior specialist
Dataset split (patient-level)	Training: 442 (80%), Hold-out test: 110 (20%)
Augmentation (training only)	Gaussian noise, random horizontal flip, Mosaic, scaling, translation
Schatzker subtype distribution	Type I: 114, Type II: 123, Type III: 94, Type IV: 71, Type V: 88, Type VI: 62

Furthermore, compared to the original YOLOv8n-seg model, the improved YOLOv8n-seg-SPDConv model reduced the number of parameters and computational complexity, measured in FLOPs, by 11.3% and 6.7%, respectively. This significant reduction in parameters and computations decreases the computational power requirements of the improved model, suggesting its potential for deployment on resource-constrained platforms. The improved detection speed under the same computational constraints (RTX 3090) further supports this potential.

As shown in Table 4, the improved model significantly enhances computational efficiency while maintaining higher detection accuracy. The overall mAP50 increases from 0.893 to 0.914, while the parameter count is reduced from 3.01M to 2.64M and the computational cost drops from 8.1G to 7.3G. To validate the model's real-time inference capability, we focus on frame rate as a key performance metric: the inference speed rises substantially from 188 FPS to 226 FPS, underscoring the model's strong potential for real-time applications. Although detection accuracy for Type I and Type II fractures exhibits minor fluctuations, decreasing by 1.3% and 0.4% respectively, performance improves markedly across Type III to Type VI fractures. Notably, for the clinically critical Type IV fractures, the mAP50 gain reaches 5.0%, providing robust evidence of the model's exceptional robustness in identifying complex and challenging fracture cases.

**Table 4:** Performance comparison of the proposed model and the baseline on fracture detection.

Model		Class	All	I	Ⅱ	Ⅲ	Ⅳ	Ⅴ	Ⅵ
**YOLOv8n-seg**		Images	110	23	25	19	14	18	11
Params	FLOPs	Mask(P)	0.848	0.87	0.965	0.787	0.814	0.859	0.79
LOPs	8.1G	R	0.842	0.918	0.904	0.86	0.759	0.827	0.785
FPS	188	mAP50	0.893	0.941	0.956	0.861	0.818	0.916	0.869
		mAP50–95	0.82	0.869	0.872	0.813	0.752	0.826	0.789
**YOLOv8n-seg-SPDConv**		Images	110	23	25	19	14	18	11
Params	2.64M	Mask(P)	0.872	0.831	0.96	0.886	0.87	0.967	0.721
FLOPs	7.3G	R	0.875	0.918	0.915	0.867	0.793	0.788	0.969
FPS	226	mAP50	0.914	0.928	0.952	0.929	0.859	0.925	0.893
		mAP50–95	0.837	0.854	0.879	0.859	0.789	0.819	0.822

Figure 5, the confusion matrix, visually presents the classification results of the six fracture types by the models before and after improvement. The performance of the YOLOv8-seg and the improved YOLOv8n-seg-SPDConv algorithm was evaluated on the hold-out test set of 110 images. Among the six analyzed types, the original YOLOv8n-seg model achieved correct recognition rates of 86%, 94%, 87%, 72%, 78%, and 79% for the six type samples, respectively. The improved YOLOv8n-seg-SPDConv showed reduced precision for Type II and Type V categories, at 87% and 70% respectively, while its detection accuracy for Type III showed no significant change. Its detection accuracy for Type I, Type IV, and Type VI improved to 92%, 76%, and 96%, respectively. This indicates that the SPDConv module added in this study enhances the model's ability to extract certain features from CT images, although it also leads to the loss of some individual features. Overall, however, the detection performance is improved.

**Figure 5:**
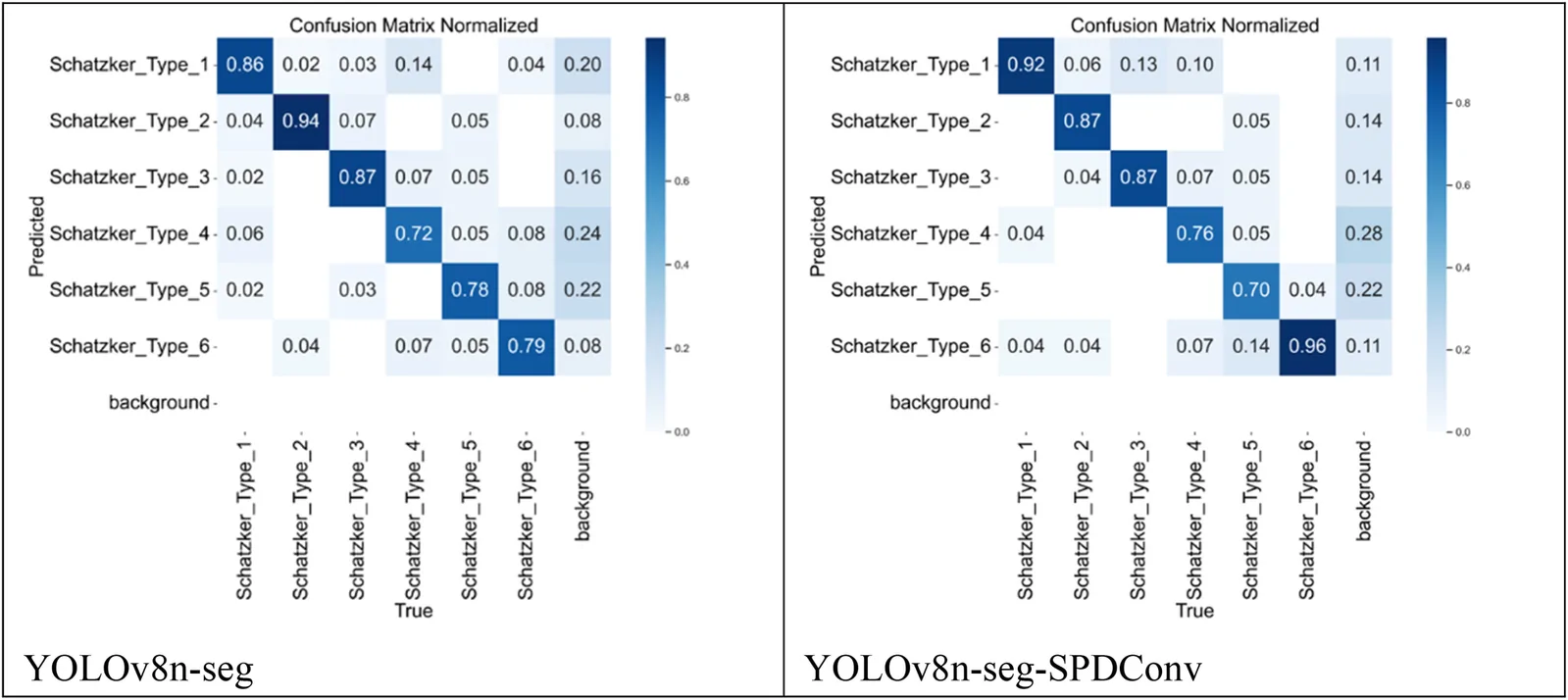
Comparison of Normalized Confusion Matrices.

Figure 6 displays the training set data, including the number of instances per category, the size and count of bounding boxes, the location of center points relative to the entire image, and the aspect ratios of the targets within the images. The top-left subplot shows the instance count for each category in the training set, i.e., how many times each category appears across all training images. The dataset used in this study has a slightly uneven distribution of samples across the six target types, which can inform subsequent understanding of the sample quantities for each class and provide a basis for data augmentation. The top-right subplot presents an overlay of all category bounding boxes aligned with the image center as the origin, offering an intuitive view of the shape, size, and approximate positional distribution of the various bounding boxes. The bottom-left subplot indicates the location of center points relative to the entire image, allowing for further analysis of the spatial location preference of defects or targets within the images. The bottom-right subplot shows the height-to-width ratios of the targets relative to the entire image, enabling further analysis of the size and aspect ratio distribution of the defect bounding boxes.

**Figure 6:**
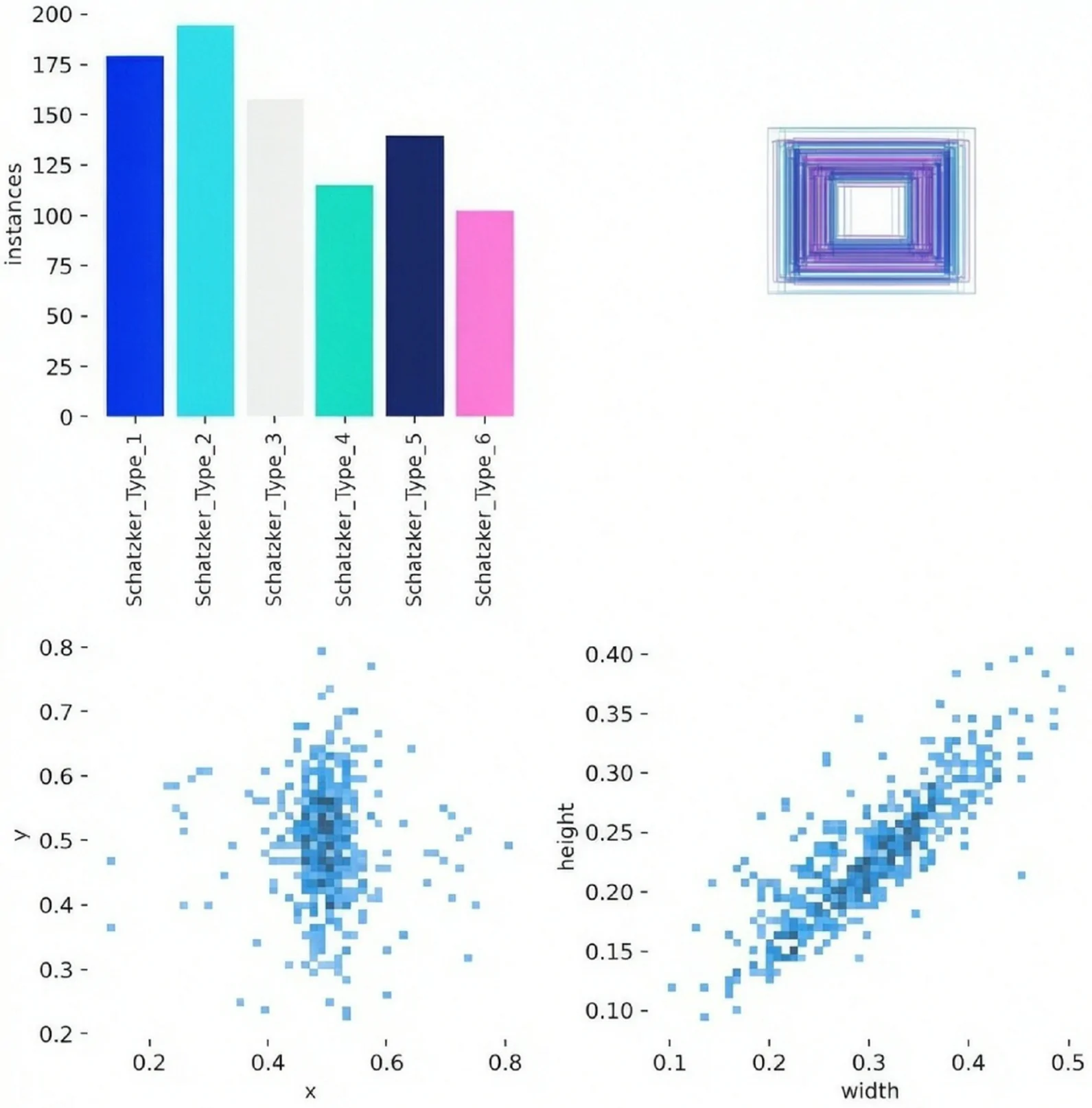
Label quantity and distribution.

Figure 7 illustrates the relationship between the horizontal and vertical coordinates of the center points and the bounding box dimensions. It shows the correlations between several key attributes of the annotated bounding boxes in the dataset. These attributes typically include the normalized coordinates (x, y) of the bounding box center, as well as its normalized width and height. The diagonal of the matrix displays one-dimensional histograms for each attribute, representing the data distribution of that individual attribute. The off-diagonal plots are two-dimensional scatter plots between two different attributes, demonstrating their interrelationships, where darker colors indicate a higher density of data points.

**Figure 7:**
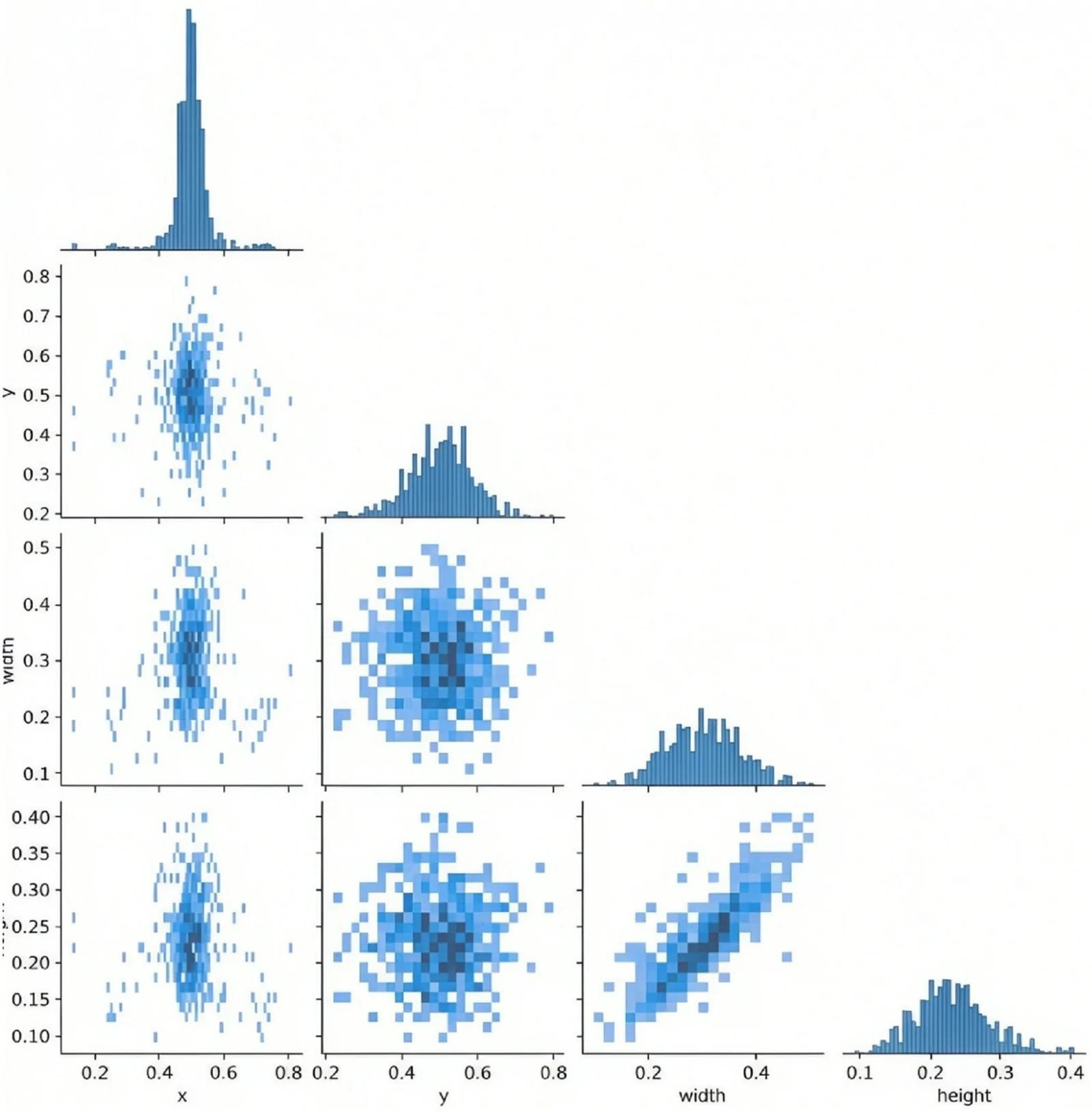
Target size distribution relationship diagram.

Figure 8 presents the loss variation curves as well as the curves for metrics such as precision and recall during the model training process. The curves showing a downward trend in Figure 8 represent the loss variation. The model uses loss functions to evaluate the discrepancy between predicted and actual values. These loss functions significantly influence the model's performance as they guide the training process towards more accurate predictions. The loss functions are divided into two parts: classification and regression. The classification loss function, cls_loss (Classification Loss), employs Binary Cross-Entropy Loss to calculate the loss between the predicted and true class labels. A lower classification loss indicates that detected objects are more precisely classified into their respective categories, ensuring the model accurately identifies the types of objects present. The regression branch uses dfl_loss (Distributed Focal Loss) and box_loss (Bounding Box Loss). The box_loss calculates the loss for the predicted bounding box's position and size relative to the ground truth box. This error is computed using the Intersection over Union (IoU) metric. A lower IoU value corresponds to higher localization accuracy, reflecting a more precise overlap between the predicted and ground truth bounding boxes. Overall, these loss functions are indispensable for optimizing the YOLOv8 model, thereby driving improvements in detection and classification accuracy.

**Figure 8:**
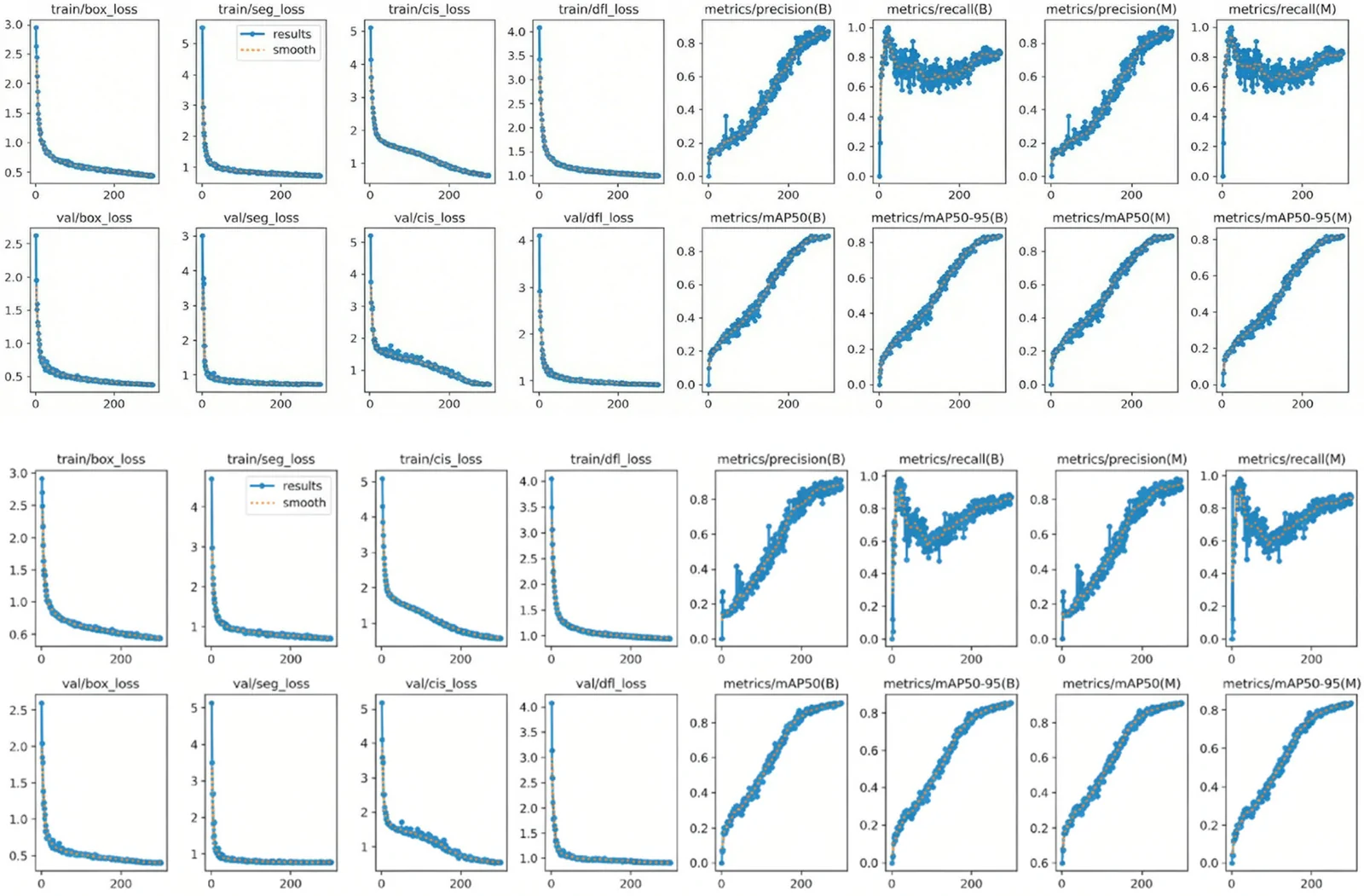
Comparison of model loss and accuracy curves.

The curves on the right side of Figure 8, which show an upward trend, represent the variation in precision, recall, mAP50, and mAP50-95 during model training. The labels “B” and “M” represent Bounding Box and Segmentation Mask, respectively. Precision is defined as a measure of the accuracy of the model's positive predictions, calculated as the ratio of correctly identified positive instances to all instances identified as positive. Higher precision indicates that the model makes fewer false positive errors, ensuring that most of its positive predictions are accurate. In contrast, Recall represents the proportion of actual positive samples that the model correctly identifies, essentially measuring the model's ability to identify all relevant instances in the dataset. It is calculated as the ratio of True Positives to the sum of True Positives and False Negatives. mAP50 and mAP50-95 represent the mean Average Precision at a confidence threshold of 0.5 and the average precision tested at confidence thresholds ranging from 0.5 to 0.95 in increments of 0.05, respectively. These metrics reflect the model's most realistic detection performance from different dimensions.

Next, a comparative analysis of the improved and original YOLOv8 models is conducted using four metrics: accuracy, recall, F1-score, and AP (Average Precision) value, to comprehensively evaluate the performance improvements across various aspects. First, regarding the accuracy for the six fracture types, in this experiment, Precision (P) represents the proportion of correctly predicted types by the model out of the total number of predicted types. Figure 9 shows the comparison chart of the *P* curves for the models before and after improvement. From the chart, it can be observed that the improved YOLOv8n-seg-SPDConv model achieves an overall mAP50 improvement from 0.893 to 0.914, with substantial gains for Types III, IV, V, and VI, while exhibiting slight reductions of −1.3% and −0.4% for Types I and II, respectively.

**Figure 9:**
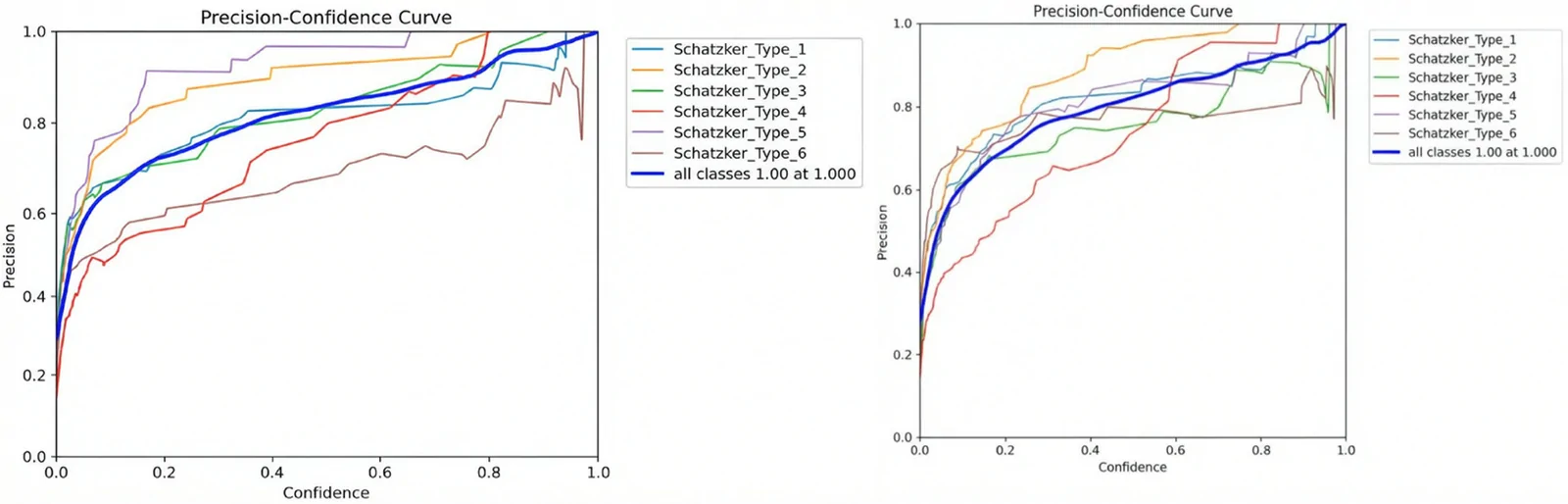
Comparison of model precision curves.

Recall (R) represents, in this experiment, the proportion of correctly predicted fracture types by the model relative to the total number of fractures. Figure 10 shows the comparison chart of the R curves for the models before and after improvement. As shown in the figure, the R curve score in the legend on the right for the improved model is 0.96, an 8% increase compared to 0.88 for the pre-improvement model. Furthermore, the R curve for each individual type shows improvement compared to the pre-improvement version. Therefore, the enhanced YOLOv8n-seg-SPDConv model achieves a certain improvement in recall.

**Figure 10:**
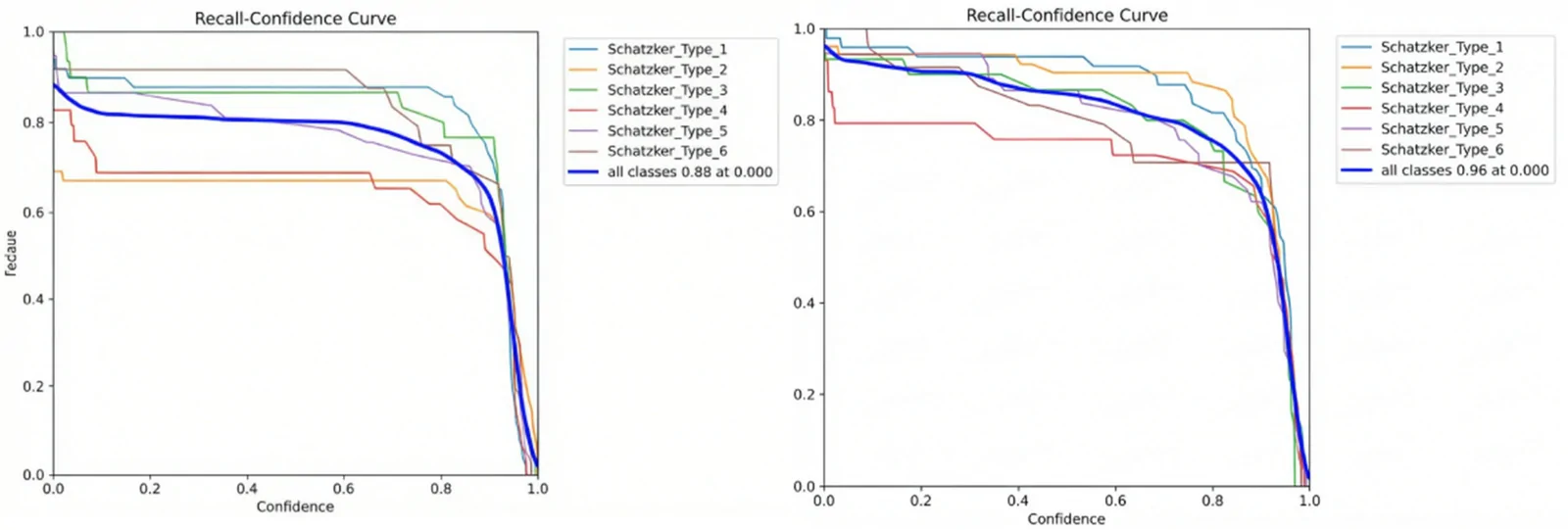
Comparison of model recall curves.

The F1-score is the harmonic mean used to calculate Precision (P) and Recall (R), integrating these two metrics. With the threshold set to 0.5, Figure 11 presents the F1 curves of the models before and after improvement. As shown in the figure, the improved model shows a decrease in the F1 curve for Type IV targets compared to the pre-improvement version. However, the F1-scores for the other five target types have slightly increased. The overall F1-score improved from 0.82 to 0.84, thereby enhancing the model's F1-score.

**Figure 11:**
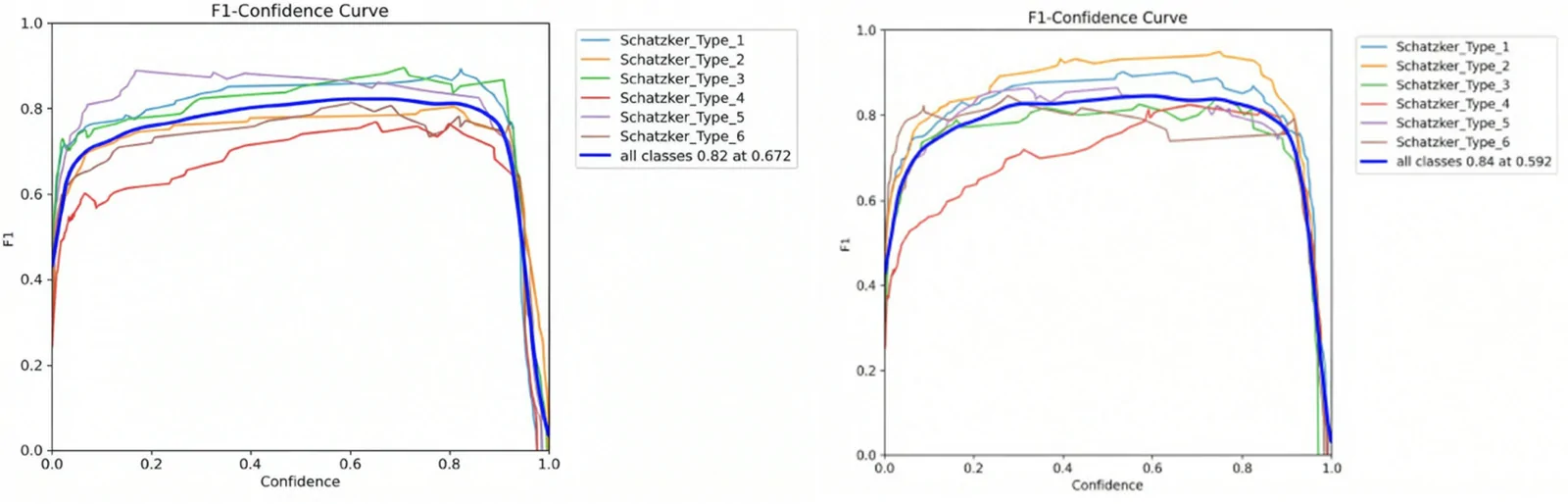
Comparison of model F1-score curves.

As the most important among the four metrics, the AP (Average Precision) value provides a more comprehensive measure of the relationship between precision and recall. Essentially, the AP value represents the area under the PR curve. A larger area indicates a higher AP value, signifying better detection accuracy for that object category. Figure 12 shows the comparison of the PR curves for the models before and after improvement. After replacing the traditional Conv modules with the SPDConv downsampling module, the improved YOLOv8n-seg-SPDConv model achieved an overall mAP50 of 0.914, compared to 0.893 for the baseline YOLOv8n-seg, representing an increase of 2.1 percentage points. For individual Schatzker subtypes, the mAP50 values changed as follows: Type I from 0.941 to 0.928 (a slight decrease of 1.3%), Type II from 0.956 to 0.952 (a slight decrease of 0.4%), Type III from 0.861 to 0.929 (an increase of 6.8%), Type IV from 0.818 to 0.859 (an increase of 4.1%), Type V from 0.916 to 0.925 (an increase of 0.9%), and Type VI from 0.869 to 0.893 (an increase of 2.4%). These results demonstrate that the integration of SPDConv generally enhances classification accuracy, with particularly notable gains for complex subtypes that rely on subtle radiographic features. The corresponding PR curves are shown in Figure 12 for visualization purposes; all quantitative metrics are reported in Table 4. The introduction of the SPDConv downsampling module led to a noticeable enhancement in the model's detection performance.

**Figure 12:**
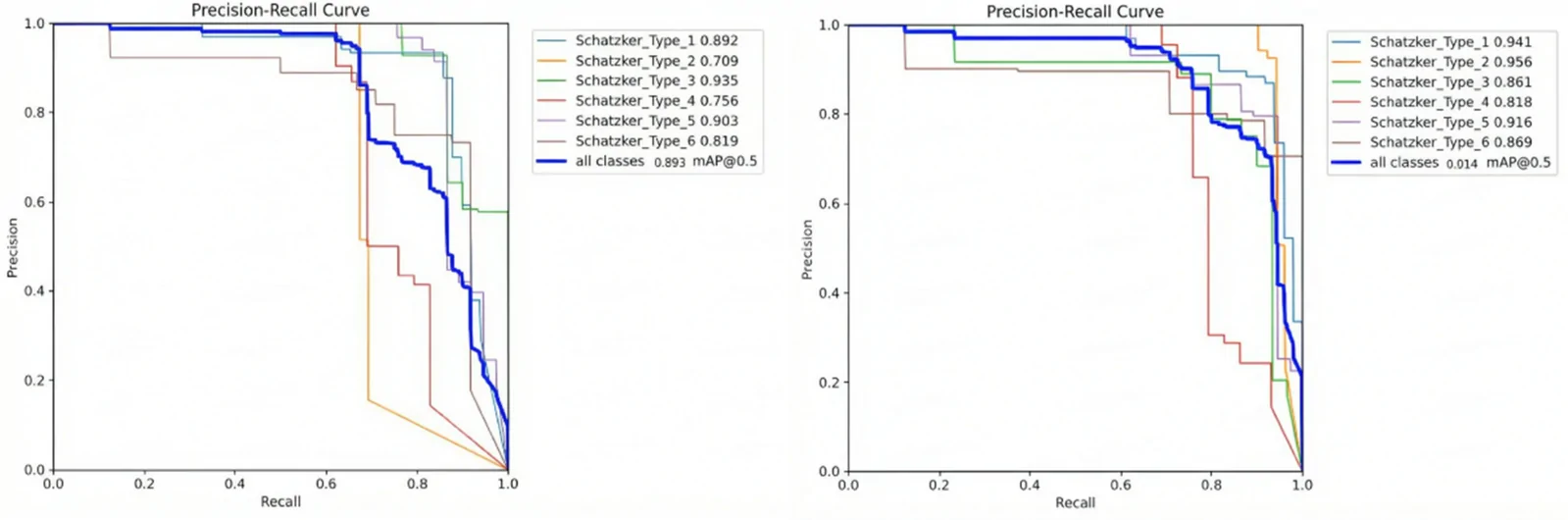
Comparison of model precision-recall curves.

Figure 13 shows the visualized recognition results of the YOLOv8n-seg model and the YOLOv8n-seg-SPDConv model. It can be observed from the figure that the improved model enhances the recognition confidence for the six fracture types: the confidence for Type I increased from 0.92 to 0.95, for Type II from 0.83 to 0.92, and for Type III from 0.87 to 0.95. The confidence levels for Types IV, V, and VI also improved accordingly. This further demonstrates that the improved model has enhanced its recognition capability for these three types of fractures.

**Figure 13:**
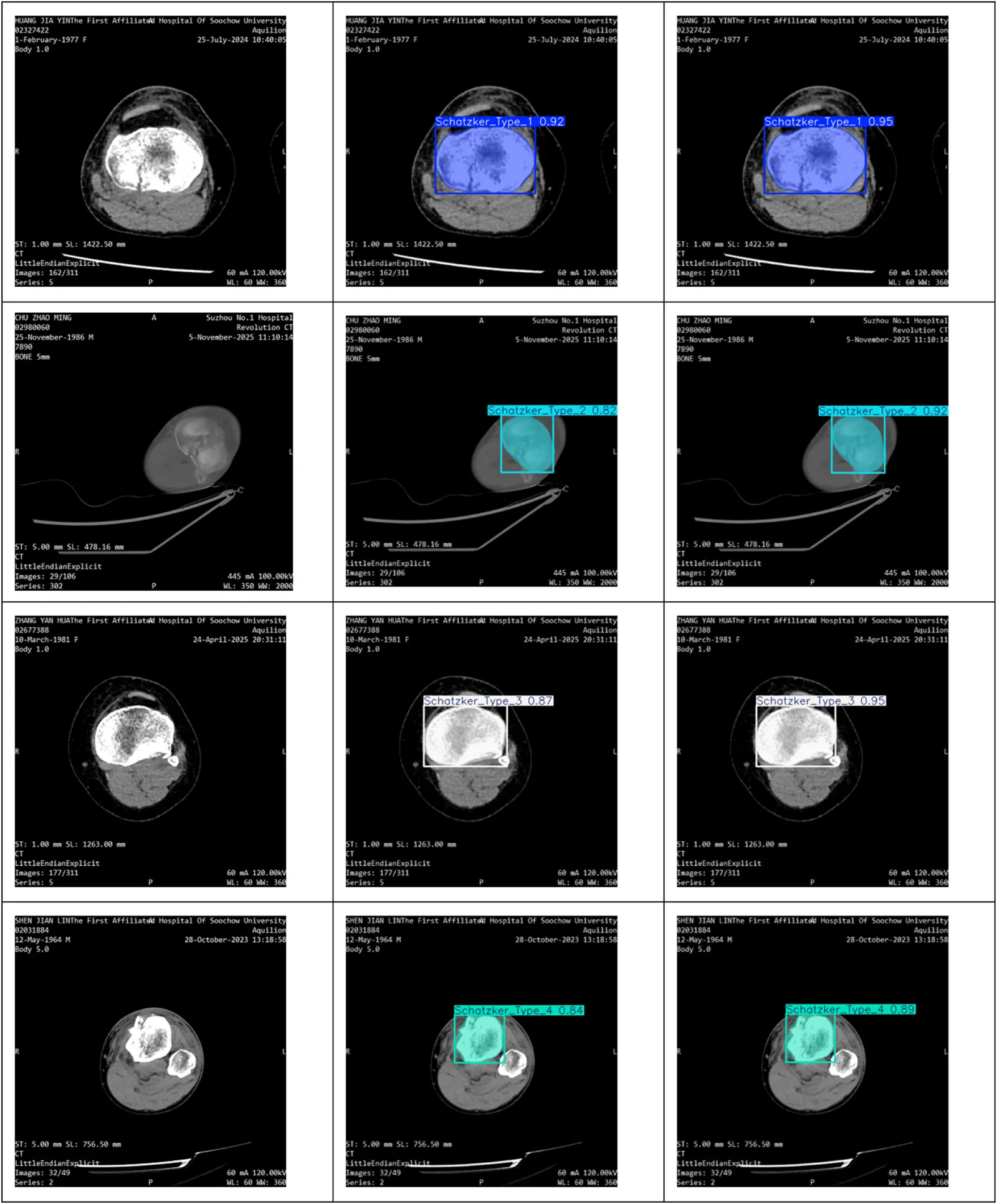
Visualization and Comparison of Model Recognition Results.

Figure 14 presents the Grad-CAM heatmaps of the baseline and proposed models. The baseline exhibits dispersed attention with background interference, whereas the proposed YOLOv8n-seg-SPDConv model concentrates on clinically relevant structures in a subtype-specific manner. For Type I, attention focuses on the lateral cortical discontinuity; for Type II, both the cortical breach and depressed fragment are activated; for Type III, the model correctly targets the subchondral step-off rather than surrounding cortex—a clinically challenging distinction; for Type IV, attention centers on the medial plateau and intercondylar eminence; and for Types V and VI, both compartments are simultaneously attended, with additional metaphyseal focus in Type VI. This confirms that the model has learned subtype-specific radiographic signatures rather than generic features, attributable to SPDConv's preservation of subtle fracture details otherwise lost in standard downsampling.

**Figure 14:**
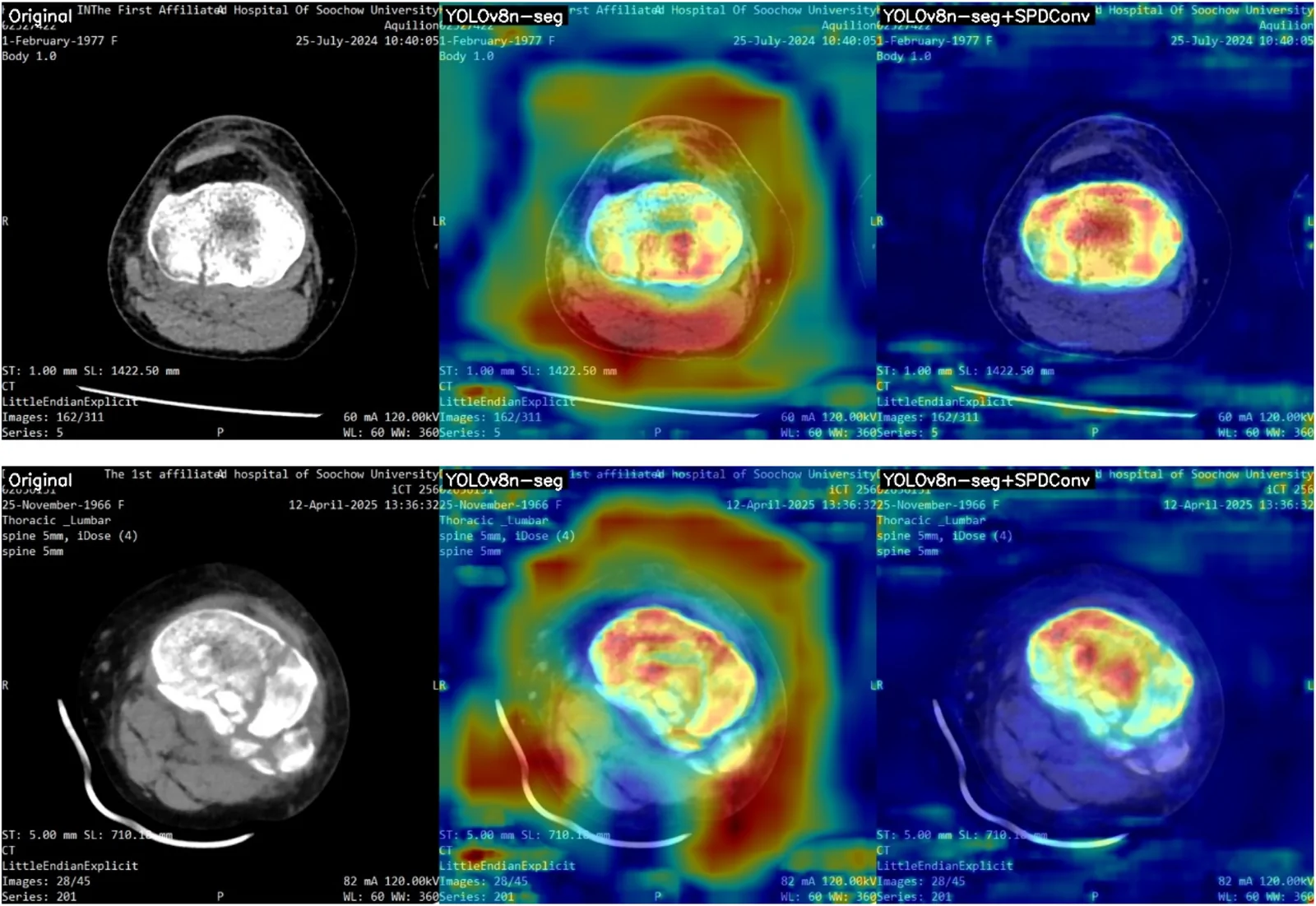
Heatmap comparison of YOLOv8n-seg and YOLOv8n-seg-SPDConv on CT images.

## 4 Discussion

It is important to clarify the specific clinical niche of the proposed model. While seasoned orthopedic surgeons can efficiently integrate multi-planar CT and radiographs to achieve high accuracy in Schatzker classification, the incremental utility of an automated tool for such experts is admittedly limited. However, the true value of this algorithm emerges in two distinct scenarios: medical pedagogy and multicenter research standardization. For junior residents and graduate students, the model acts as a “digital teaching aid” by highlighting subtle radiographic features—such as cortical breaches smaller than 2 mm—that are often missed during initial training. This may help bridge the cognitive gap between textbook descriptions and real imaging. Furthermore, in multicenter randomized controlled trials (RCTs) investigating tibial plateau fracture outcomes, inter-observer variability in manual subtyping often introduces confounding bias. Our model provides a standardized, reproducible reference to harmonize subgroup definitions across participating centers, thereby enhancing the statistical validity of comparative effectiveness analyses. Consequently, rather than serving as a diagnostic crutch for experts, this tool is designed to standardize care in primary care hospitals, expedite triage in high-throughput emergency departments lacking immediate specialist coverage, and function as a benchmark for trainee assessment.

In this study, researchers employed the YOLOv8-Seg deep learning model to classify tibial plateau fractures according to the Schatzker classification. The results showed that, compared to the original YOLOv8n-seg model, the improved YOLOv8n-seg-SPDConv model achieved an improvement in detection accuracy. By incorporating the SPDConv module, although the correct recognition rates for the six fracture types varied—with some increasing and others decreasing—the improved model's mean average precision mAP increased to 91.4%. This improvement was achieved while simultaneously reducing the model's parameter count and enhancing the detection and segmentation performance for most fracture types. A certain improvement in recall was also observed. The model's detection and recognition capabilities were thus significantly improved. Although the SPDConv module introduces additional tensor rearrangement operations compared to standard strided convolution, it effectively reduces the overall computational burden. In this study, replacing the original convolutional downsampling layers with SPDConv decreased the model parameters from 3.01 M to 2.64 M and the floating-point operations from 8.1 G to 7.3 G. More importantly, this reduction directly translated into improved inference efficiency: under identical hardware conditions on an NVIDIA RTX 3090, the average inference time per axial CT slice was shortened from 5.32 ms to 4.42 ms, representing a throughput increase of approximately 20.2%. The Space-to-Depth transformation avoids the irreversible information loss inherent in stride-2 convolutions, while the subsequent 1 × 1 convolution mitigates the slight latency potentially introduced by channel concatenation. Consequently, the YOLOv8n-seg-SPDConv model not only achieves superior accuracy but also demonstrates reduced model complexity, laying a foundation for future deployment on edge devices with limited computational resources. Actual performance on embedded hardware warrants further investigation in subsequent studies.

This study maintained rigorous monitoring for overfitting throughout the entire training process. As illustrated in Figure 8, both the classification loss and regression losses converged smoothly after approximately 180 epochs, with no significant divergence observed between the training and validation loss curves. Ultimately, the gap in mAP50 between the training and validation sets was less than 2.1%, which falls within the acceptable range for medical image segmentation tasks. To further suppress overfitting, we implemented several strategies: (1) employing diverse data augmentation techniques, including Gaussian noise injection, Mosaic augmentation, random flipping, scaling, and translation; (2) introducing a class-weighted loss function to mitigate the class imbalance across Schatzker subtypes; and (3) adopting an early stopping strategy based on validation set performance. These measures ensure that the improvement in mAP primarily stems from enhanced generalization capability rather than mere memorization of the training samples.

To validate the robustness of the proposed method, we conducted 2-fold cross-validation on the original cohort of 552 images from 552 unique patients. Dataset partitioning was performed at the patient level to prevent data leakage, and class distribution was approximately preserved across folds. In each iteration, one subset served as the training set while the other functioned as the validation set. Consistent with the main experimental protocol, Gaussian noise augmentation was applied exclusively to the training subset of each fold, while the validation subset retained only unaugmented original images. This leakage-free cross-validation framework ensured that no original image and its augmented counterpart appeared across different folds.

As summarized in Table 5, the two folds represent two independent training runs with distinct patient-wise partitions, ensuring no data leakage. Across these repeated experiments, the proposed YOLOv8n-seg-SPDConv yielded mean ± SD values of 0.867 ± 0.001 for Mask Precision, 0.865 ± 0.002 for Recall, 0.909 ± 0.004 for mAP50, and 0.837 ± 0.006 for mAP50-95. These results consistently outperformed the baseline YOLOv8n-seg (0.836 ± 0.002, 0.825 ± 0.001, 0.885 ± 0.004, and 0.821 ± 0.006, respectively). Crucially, the low standard deviations confirm that the performance gain introduced by the SPDConv module is stable and reproducible, rather than derived from stochastic variation in a single training run.

**Table 5:** Results of the 2-fold cross-validation.

Group	Model	Mask(P)	R	mAP0.5	mAP0.5:0.95
Group1	YOLOv8n-seg	0.834	0.826	0.882	0.816
	YOLOv8n-seg-SPDConv	0.866	0.863	0.906	0.833
Group2	YOLOv8n-seg	0.837	0.824	0.888	0.825
	YOLOv8n-seg-SPDConv	0.868	0.867	0.911	0.841
Average	YOLOv8n-seg	0.836 ± 0.002	0.825 ± 0.001	0.885 ± 0.004	0.821 ± 0.006
	YOLOv8n-seg-SPDConv	0.867 ± 0.001	0.865 ± 0.002	0.909 ± 0.004	0.837 ± 0.006

Our results demonstrate that applying deep learning models addresses specific inherent challenges in CT imaging of tibial plateau fractures, effectively enhancing the accuracy of fracture classification in clinical image analysis. For instance, distinguishing between Schatzker Type II and Type III fractures—which appear morphologically similar—hinges on identifying subtle cortical disruptions and the presence of a split component. On axial CT slices, these features may span only a few pixels. Similarly, evaluating Type IV fractures requires the precise identification of fracture lines extending toward the intercondylar eminence. Traditional convolutional downsampling tends to further smooth out these weak signal differentials, preventing the model from perceiving the discontinuity of the articular surface and leading to missed diagnoses of depressions. By leveraging the SPDConv module, these critical details are preserved by transforming spatial information into the channel dimension, thereby retaining sub-pixel textures and serrated fracture margins. This precise preservation of subtle linear fracture features explains why our model exhibited significant performance improvements in detecting Type I and Type IV fractures.

While the integration of the SPDConv module yields overall performance gains, it introduces specific algorithmic trade-offs that warrant explicit discussion. First, SPDConv increases intermediate channel dimensions (from *C*to *4C*prior to the subsequent 1 × 1 convolution), which theoretically elevates sensitivity to high-frequency noise, such as metal streak artifacts from internal fixation hardware or quantum mottle in low-dose CT acquisitions. In this study, however, Gaussian noise augmentation during training effectively mitigated this risk, as evidenced by stable mAP50-95 values across all subtypes and the absence of spurious edge detections in non-osseous regions on heatmap visualization (Figure 14). Second, the channel expansion inherent to SPDConv may induce minor feature response shifts when processing high-contrast, sharply delineated linear structures. This mechanism explains the negligible mAP50 reductions observed in Type I (−1.3%) and Type II (−0.4%) fractures, whose defining cortical disruptions are already sufficiently captured by conventional strided convolution in the baseline model. The confusion matrix (Figure 5) further corroborates this interpretation: misclassifications for Type I predominantly involve confusion with Type II (both lateral plateau injuries), rather than random assignment, indicating that SPDConv does not introduce systematic bias but rather induces subtle boundary response variations for already well-defined fracture lines. Importantly, these marginal decrements are offset by substantial gains in subtypes reliant on low-contrast morphological details, confirming that the algorithmic modifications align with the intrinsic imaging characteristics of tibial plateau fractures.

Notably, Schatzker type III exhibited relatively large performance fluctuations compared with other subtypes. Unlike types I and II, which involve cortical disruption, type III fractures are characterized solely by central depression of the lateral tibial plateau without peripheral cortical discontinuity. On axial CT images, the hallmark of type III is a subtle step-off of the subchondral cortex, which is highly susceptible to partial volume averaging artifacts and demonstrates significant overlap with the physiological undulations of the tibial plateau. This inherent ambiguity in imaging phenotypes creates diagnostic uncertainty even among experienced radiologists when differentiating true pathological depression from pseudodepression. From a dataset perspective, type III had the second smallest sample size among the six subtypes, limiting the model's exposure to morphological variability. Furthermore, the depression in type III cases is often shallow, producing weak radiological signals that are readily obscured during deep feature extraction. Although SPDConv effectively preserves fine-grained spatial information, its advantages are difficult to fully leverage when imaging phenotypes closely resemble normal anatomy and ground-truth boundaries are intrinsically ambiguous. Further analysis revealed that type III cases in our cohort predominantly involved shallow depressions adjacent to the intercondylar eminence. This region is anatomically challenging due to its naturally high curvature and substantial inter-individual variation, further complicating automated recognition. Additionally, the absence of high-contrast linear fracture lines deprives the model of the salient edge features that facilitate the classification of other subtypes. Collectively, these imaging and anatomical characteristics render type III the most challenging category for both conventional diagnostic methods and deep learning-based classifiers. To address this, future iterations of our dataset will prioritize multi-planar reformation and three-dimensional CT volumetric data, as sagittal and coronal reconstructions more clearly delineate central depressions. Nevertheless, our findings robustly demonstrate that deep learning holds significant potential for the automated classification of tibial plateau fractures and may serve as a reliable and efficient adjunctive diagnostic tool for clinicians.

Although the improved model exhibited a slight decrease in mAP50 for Type I and Type II fractures, we attribute this to the architectural characteristics of the SPDConv module and the specific imaging features of these fracture types. The fracture lines in Types I and II are typically sharp and exhibit high contrast, features that are adequately captured by the standard strided convolution in the baseline model. In contrast, SPDConv preserves fine details by mapping spatial information into the channel dimension; however, when processing such “strong linear, high-contrast” features, this mechanism may induce minor shifts in feature responses due to channel redundancy or the over-smoothing of specific textures. Furthermore, as Types I and II constitute the majority of the dataset, the baseline model was already highly optimized for these classes, leaving limited room for improvement. Consequently, the feature distribution shift introduced by SPDConv may have triggered a slight tendency toward overfitting in these highly fitted regions. Nevertheless, the magnitude of this decline is negligible and falls within clinically acceptable margins. Crucially, the model demonstrated significant performance gains for Types III and IV—categories characterized by smaller sample sizes and subtler radiographic features—indicating that the primary advantage of SPDConv lies in its enhanced capability to recognize challenging cases.

Integrating this model into the clinical workflow offers multiple potential benefits. The model can complete analysis within seconds, which may help reduce diagnostic time and potentially minimize subjective variability caused by factors like differences in physician experience or fatigue, particularly in primary care hospitals or emergency departments. Accurate Schatzker classification is a critical basis for formulating surgical plans, such as the choice of internal fixation method and surgical approach (30). This system can provide reliable classification references for doctors, especially junior physicians, thereby strengthening decision support. By facilitating earlier and more accurate diagnosis and classification, it aids in developing more appropriate treatment plans. This may reduce the risk of complications such as malunion and post-traumatic arthritis, ultimately improving long-term patient rehabilitation outcomes.

Although this study confirms that applying the YOLOv8-Seg deep learning model to tibial plateau fracture image analysis yields favorable results, the model still has certain limitations. First, the sample size remains modest compared with similar studies in the field (20, 21). With 552 images distributed across six fracture subtypes, certain minority classes contain fewer than 75 images each. This limited data volume may constrain the model's ability to learn comprehensive and generalizable features, potentially leading to unstable performance when encountering rare or exceptionally complex fracture patterns. Formal hyperparameter sensitivity analysis was not performed; the selected configuration follows established YOLOv8-seg defaults and is supported by stable cross-validation results (Table 5) and training dynamics (Figure 8).

Second, this study utilized only the Schatzker classification system. While widely used, this system is also acknowledged to have limitations, such as complexity, in practical application (7, 31, 32). Other important systems like the AO/OTA classification and the three-column theory are also used in clinical practice. Different classification standards have their own emphases and are suitable for different clinical scenarios. This study has not yet explored the model's capability to handle multiple classification standards simultaneously, which to some extent limits the universality of its clinical application.

Third, the 2-fold cross-validation employed in this study offers limited statistical rigor. Although cross-validation was conducted at the patient level on the original 552-image cohort—with augmentation restricted to training subsets to prevent data leakage—the 2-fold design may not adequately capture performance variance across diverse patient populations, particularly for minority subtypes. Higher-order k-fold cross-validation would provide a more robust estimate of model generalizability but was not feasible given the current dataset scale. This constraint is noted as a limitation, and future multicenter expansions will enable more rigorous validation frameworks.

Fourth, the generalizability of the proposed model remains unvalidated. All data were collected from a single institution with a limited range of CT scanners and protocols. The model has not been tested on external datasets from other hospitals, different scanner manufacturers, or diverse patient populations. Additionally, the exclusive use of axial CT images may limit applicability in settings where multiplanar reconstructions are routinely employed. Multi-institutional external validation is therefore necessary to assess the model's robustness across heterogeneous clinical environments.

Fifth, although a rigorous dual-expert consensus protocol was adopted to establish the gold standard, we did not quantitatively report inter-observer agreement (e.g., Cohen's kappa) prior to consensus, as independent pre-consensus classifications were not separately archived. Future studies should retain independent annotations from each expert to enable formal agreement analysis and further strengthen the validation of the reference standard.

Based on the achievements and limitations of the current research, future work will focus on the following directions: expanding the dataset scale by incorporating more diverse data from multiple medical institutions; conducting prospective clinical studies to evaluate the model's effectiveness in real clinical environments; exploring the interpretability of the model to make its decision-making process more transparent to clinicians. The ultimate goal is to develop it into clinical auxiliary diagnostic software integrated into Picture Archiving and Communication Systems. This would genuinely empower the precise diagnosis and treatment of tibial plateau fractures, enabling real-time auxiliary diagnosis in settings like emergency departments or orthopaedic clinics, optimizing diagnosis and treatment workflows, and improving long-term functional prognosis for patients.

## 5 Conclusion

This study addresses the challenges in classifying tibial plateau fractures. With the increasing number of patients with such fractures in recent years, the importance of related research has become more prominent. By utilizing advanced deep learning methods, the model developed in this study improves the accuracy and efficiency of clinical image diagnosis, aiding clinicians in making better decisions and reducing their workload. Furthermore, this research is instrumental in helping design improved treatment plans for patients, thereby mitigating the risks associated with misdiagnosis.

Currently, the study also has limitations, such as a limited sample size, a single data source, and reliance on only one classification standard. Future research plans to address these limitations by expanding the dataset, conducting multi-center prospective clinical validation, and exploring the model's compatibility with multiple classification standards. These steps aim to further enhance the model's generalization capability and clinical practical value.

## Data Availability

The raw data supporting the conclusions of this article will be made available by the authors, without undue reservation.
